# A review of animal models utilized in preclinical studies of approved gene therapy products: trends and insights

**DOI:** 10.1186/s42826-024-00195-6

**Published:** 2024-04-22

**Authors:** Parham Soufizadeh, Vahid Mansouri, Naser Ahmadbeigi

**Affiliations:** 1 Gene Therapy Research Center, Digestive Diseases Research Institute, Shariati Hospital, Tehran University of Medical Sciences, Tehran, Iran; 2 Biomedical Research Institute, University of Tehran, Tehran, Iran

**Keywords:** Animal model, Preclinical study, Gene therapy, Trends

## Abstract

Scientific progress heavily relies on rigorous research, adherence to scientific standards, and transparent reporting. Animal models play a crucial role in advancing biomedical research, especially in the field of gene therapy. Animal models are vital tools in preclinical research, allowing scientists to predict outcomes and understand complex biological processes. The selection of appropriate animal models is critical, considering factors such as physiological and pathophysiological similarities, availability, and ethical considerations. Animal models continue to be indispensable tools in preclinical gene therapy research. Advancements in genetic engineering and model selection have improved the fidelity and relevance of these models. As gene therapy research progresses, careful consideration of animal models and transparent reporting will contribute to the development of effective therapies for various genetic disorders and diseases. This comprehensive review explores the use of animal models in preclinical gene therapy studies for approved products up to September 2023. The study encompasses 47 approved gene therapy products, with a focus on preclinical trials. This comprehensive analysis serves as a valuable reference for researchers in the gene therapy field, aiding in the selection of suitable animal models for their preclinical investigations.

## Background

In the realm of gene therapy, a pivotal moment arrived with Paul Berg’s groundbreaking identification of the first recombinant DNA in 1972 [1]. This achievement not only marked a significant milestone but also served as the catalyst for a series of transformative breakthroughs in the field. Berg’s discovery fundamentally altered the landscape of genetic research, opening doors to novel therapeutic possibilities and paving the way for a new era of innovation and advancements in genetic engineering and gene therapy. Given the accelerated development of gene therapy products throughout the past century, this trend is anticipated to persist into the future [2], with a substantial portion of therapeutic inquiries focusing on preclinical investigations.

The principal objective of this comprehensive review article is to scrutinize and interpret preclinical research about gene therapy products that have garnered current approval and are presently administered to patients. This endeavour aspires to serve as an invaluable reference for researchers embarking on endeavours within the realm of gene therapy, seeking suitable animal models to facilitate their scientific undertakings.

## Main text

### The importance of preclinical studies in gene therapy clinical trials

Preclinical studies in the field of gene therapy play a pivotal role in advancing our understanding of genetic diseases and developing potential treatments. Additionally, all scientific progress and development are intricately intertwined with prior research endeavours. For scientific investigations to pave the way for significant advancements, they should embody three distinct attributes: (1) Adherence to Scientific Standards: The formulation and documentation of a study must strictly adhere to established scientific norms and guidelines. (2) Rigorous Parameterization in Animal Studies: In the realm of animal studies, meticulous attention to parameters is essential to ensure the reliability and validity of such investigations. (3) Transparent and Comprehensive Reporting: Researchers should exert utmost diligence in generating a report that is transparent, comprehensive, and credible in its entirety [3]. When these fundamental principles are observed in animal studies, they hold the potential to yield profound implications for the development of therapeutic products and our comprehension of disease pathophysiology. For instance, one of the most significant advantages of preclinical gene therapy studies is their ability to address diseases that lack effective avenues for investigation in human subjects, especially in the case of rare genetic diseases. In such instances, the creation of a standardized disease model not only facilitates the examination of all disease stages but also allows for elucidating the initial pathophysiological processes, even before the onset of clinical manifestations. Furthermore, some of these models elucidate genetic interrelationships, thereby uncovering potential modifier genes, a pursuit unfeasible within the confines of human subjects [4].

However, it is important to note that the success of preclinical gene therapy studies heavily relies on their adherence to scientific rigor, transparency, and meticulous reporting. The lack of these attributes can lead to issues such as irreproducibility and non-reproducibility, which hinder progress in the field [5–12]. This predicament often arises due to incomplete or inaccurate descriptions within research protocols, encompassing the allocation of animals among disparate study groups and the criteria underpinning the formation of said groups [11]. In addition to the formidable challenge of irreproducibility, another substantial hurdle resides in the discordance between the outcomes of animal studies and the results obtained from clinical trials. For example, clinical trials investigating stroke frequently yield results that diverge markedly from those generated in preclinical studies of the same condition. Root causes for this dissonance have been traced to the inability of any animal model to faithfully replicate the intricacies of human patients and the absence of robust, well-documented methodologies in the conduct of animal studies [13].

Considering the aforementioned quandaries, animal studies that yield congruent results in clinical trials can furnish superior methodologies for advancing subsequent investigations in related domains.

### Animal models in gene therapy

The use of animal models in biomedical research, including gene therapy, is essential for gaining insights into complex biological systems and predicting the behaviour of interventions under specific conditions. These models serve as invaluable tools for researchers and can broadly be categorized into two primary functions: elucidating a system or process and predicting the behaviour of the target in question [14]. The concept of analogical reasoning, as initially introduced by Kant in the “Critique of Judgment”, posits that qualitative similarities between entities can be leveraged to forecast causal relationships, even in the presence of disparities [14]. With the advent of this concept, the application of models expanded across various scientific disciplines [15]. For instance, in the field of shipbuilding, scaled-down models are scrutinized to assess their designs, as hydrodynamics principles remain consistent, independent of scale. Conversely, in the biomedical sciences, including gene therapy, scalability lacks relevance [14] due to the diverse physical and behavioural attributes of organisms that impede such modelling. According to August Krogh’s principle, “For many problems, there is an animal on which it can be most conveniently studied” [16]. In biological sciences, the concept of analogy has supplanted scale, and its widespread applicability is attributed to the notion of “unity in diversity”, signifying fundamental relationships among organisms in terms of evolution and development [14]. Consequently, numerous animal models, notably laboratory animals such as mice, have been harnessed in diverse biological research endeavours.

Until 1980, mouse models predominantly comprised wild-type or spontaneously mutant species. Progress in fields such as chemotherapy and DNA-damaging agents owes much to the utilization of these animal models. Over the last four decades, a multitude of models catering to distinct objectives have emerged, thereby fostering advancements across various domains of biological science [17]. In recent decades, the significance of animal models has burgeoned due to the expansion of therapeutic product development, increased preclinical testing, and clinical trials. Foretelling therapeutic and safety outcomes in humans now constitutes the primary objective of experiments conducted before these products enter development, heavily contingent upon the judicious utilization of animal models [18].

The classification of animal models in the gene therapy era poses a formidable challenge, given their rapid proliferation and ongoing evolution. Moreover, diverse types of animal models each serve specific purposes, underscoring the critical importance of selecting the ideal model aligned with the research objectives. Meticulous model selection is imperative, as an erroneous choice can lead to inefficient resource allocation, ethical quandaries, and the generation of erroneous and unreliable scientific findings, potentially perpetuating inaccuracies in future experiments [19]. A 1985 NRC (National Research Council) report outlined various factors for the judicious selection of an appropriate animal model [14]. Paramount among these factors is the consideration of physiological and pathophysiological similarities between the model and the target of research. Additionally, the model’s capability to emulate desired conditions, such as disease-like states similar to those in the target (e.g., humans), warrants due consideration. Factors encompassing the model’s availability, size, lifespan, and others also play integral roles in this selection process [20]. Furthermore, individuals should be vigilant about potential mental and unconscious biases when selecting models, as familiarity or ease of use may unduly influence their choices [14].

One approach to mitigate the risk of inappropriate model selection involves the utilization of models specifically engineered for diverse conditions, such as genetically modified or humanized models closely mirroring human physiology in many aspects [21]. These models have witnessed substantial growth and find widespread application in research. Additionally, there are instances where a single animal model may prove inadequate to fulfill research objectives, necessitating the concurrent use of multiple models to ensure reliable and desired research outcomes [22]. Despite the multifaceted aspects elucidated concerning animal models, they are not the panacea for generalizing results and making biomedical predictions. It is essential to recognize that while alternatives to animal models have advanced significantly, they remain the sole practical choice for numerous experiments pertinent to human-related investigations. Numerous studies underscore that, notwithstanding their limitations, animal models persist as the primary resource for a multitude of experiments involving human subjects [14].

### Preclinical gene therapy studies

In this comprehensive analysis, a total of 47 approved gene therapy products, spanning from the inaugural approval of Vitravene to the latest sanctioned product as of September 2023, were meticulously scrutinized. The principal aim of this investigation entailed the retrieval of peer-reviewed publications about the preclinical trials of each product. This endeavour encompassed an extensive exploration through various means, including the pursuit of literature referencing the product’s generic nomenclature, the examination of the backgrounds of the contributing authors, and the scrutiny of pertinent articles from diverse sources. In some instances, official documents released by the regulatory bodies responsible for product approval were also consulted. In certain cases, regrettably, no accessible information concerning preclinical drug investigations was ascertainable. It is noteworthy that references cited within articles linked to the product under study were occasionally examined, even if the specific product was not explicitly mentioned therein. Furthermore, it should be noted that in several instances, multiple animal models were employed for the preclinical assessments. Additionally, a prevalent feature across the majority of these investigations was the reliance on common laboratory animals for safety and pharmacological studies, albeit without explicit specification.

The aggregate findings of this extensive inquiry yielded a corpus of 74 distinct animal models. The classification of animal models can be approached through various taxonomies, such as that delineated by Prabhakar, which delineates four primary categories: inbred strains, disease induction, xenograft, and genetically engineered models. Inbreeding has classically been used to obtain genetically homogeneous animals. Disease induction models are very commonly used to examine pathophysiology and drug development. Disease induction animal models involve manipulating animals to study and replicate specific diseases for research purposes. Xenograft animal models involve transplanting human cells, tissues, or tumour s into immunodeficient animals to study disease and treatment responses. Genetically engineered models are developed by altering the genetic composition of an animal by mutating, deleting, or overexpressing a targeted gene [23].

**Fig. 1:**
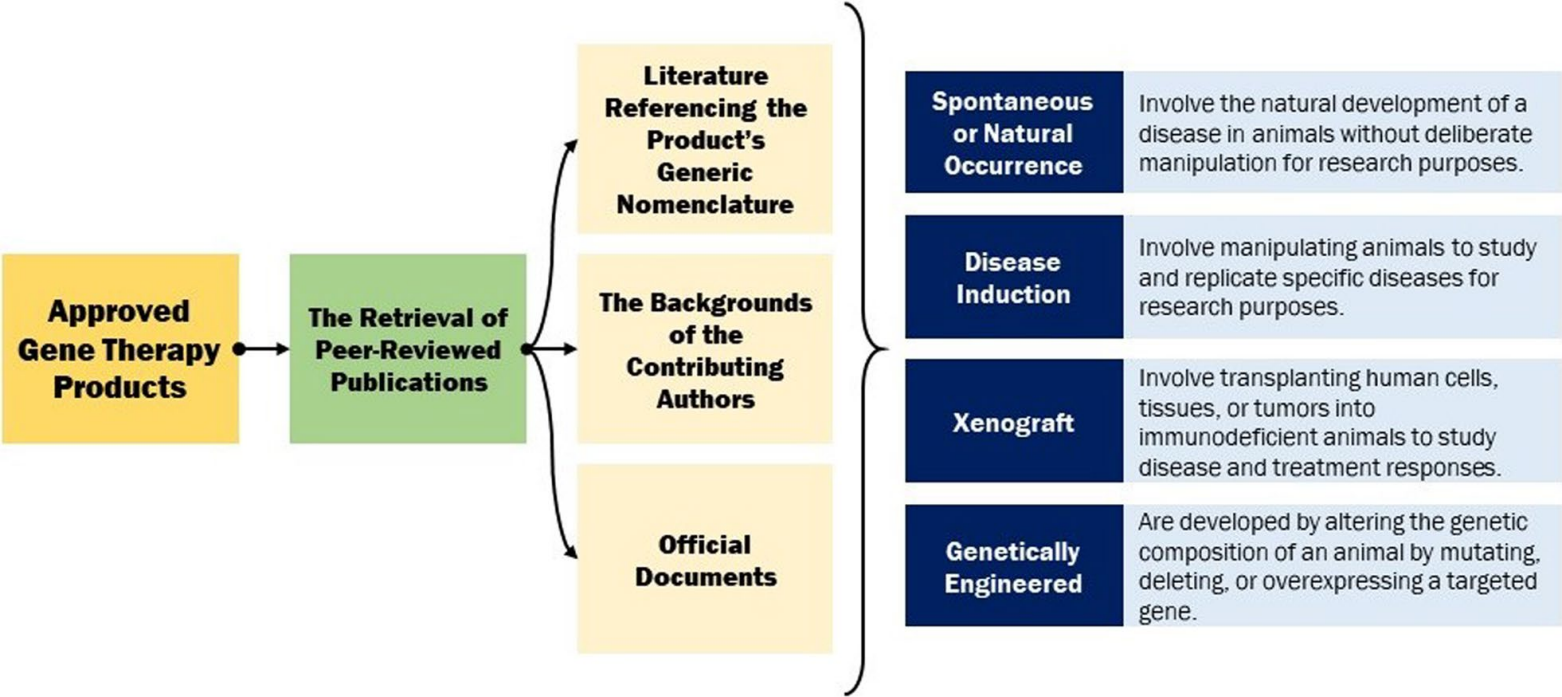
Overview of the study. In this study, by reviewing the available documents about the approved gene therapy products, the animal models used are categorized into 4 main sections

In alignment with the research objectives of this study, the “inbred” category within Prabhakar’s taxonomy was omitted, and a novel category denominated “spontaneous or natural occurrence” was introduced. Spontaneous or naturally occurring animal models involve the natural development of a disease in animals without deliberate manipulation for research purposes [24]. Consequently, the animal models under examination were categorized into four principal groups: disease induction, xenograft, genetically engineered, and spontaneous. In instances where the available information regarding the nature of the animal model utilized in the preclinical investigations of the product was indistinct or inadequately documented, such instances were classified as not applicable or N/A. It is pertinent to highlight that certain animal models were the product of mating between two animals with predetermined genetic attributes. In cases where the parentage of such models was naturally occurring, they were categorized as spontaneous. Conversely, if one or both progenitors had undergone genetic manipulation, their progeny were categorized as genetically engineered **(**Fig. 1**)**.

**Fig. 2:**
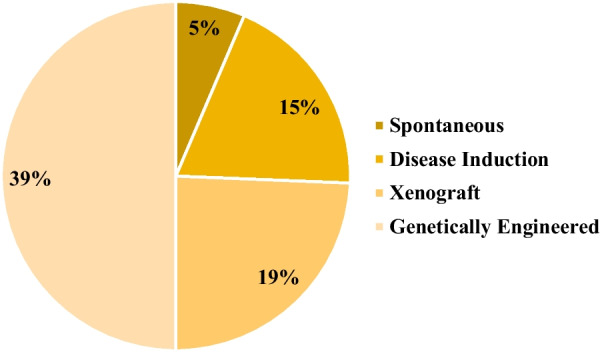
Preclinical studies based on the category of animal model development

**Fig. 3:**
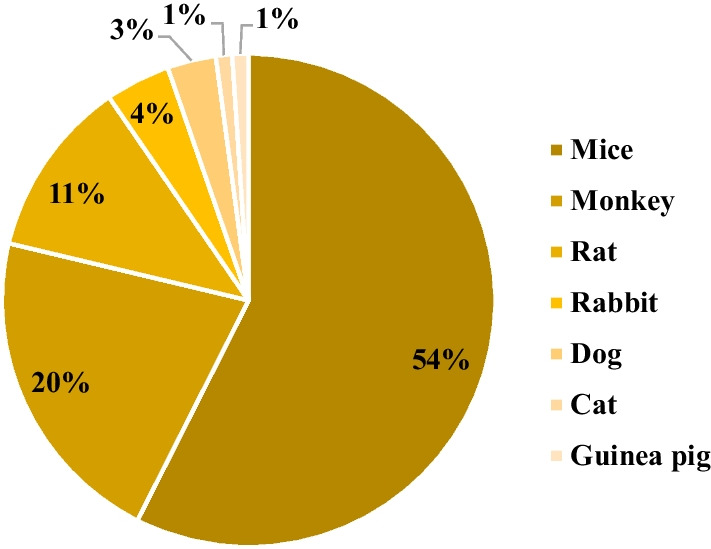
Preclinical studies based on the species of animal model

In the broader context, the analysis revealed that the genetically engineered category accounted for 39% of the identified animal models, followed by xenograft, disease induction, and spontaneous categories, with contributions of 19%, 15%, and 5%, respectively (Fig. 2). Additionally, 22% of the discerned animal models fell into the N/A category. Among the gamut of models scrutinized, mice emerged as the most frequently employed animal species, constituting 54% of the studies. Nonhuman primates claimed the second position, representing 20% of the investigated studies. Notably, other species were also incorporated into these investigations, including rats, rabbits, dogs, guinea pigs, and cats. A total of 6% of the studies did not involve the utilization of animal models (Fig. 3).

Furthermore, a granular examination of each category revealed distinctive utilization patterns. In the genetically engineered category, mice predominated, accounting for 79% of the animal species used, trailed by rats at 17%, and nonhuman primates at 7%. In the disease induction category, nonhuman primates emerged as the most frequently employed species, constituting 37% of the cases, with mice and rabbits equally sharing an 18% representation, while rats accounted for 27%. The xenograft category was overwhelmingly dominated by mice, comprising 93% of the animal species employed, with the residual 7% being nonhuman primates. In the spontaneous category, dogs featured 50% of the cases, followed by cats and mice, both with equal prevalence. Consequently, mice held sway in the genetically engineered and xenograft categories, while monkeys took precedence in the disease induction category, albeit with a caveat that 53% of the instances involving monkeys were categorized as uncertain, lacking substantive information regarding their role in the conducted studies. In the genetically engineered and disease induction categories, rats featured prominently (Table 1).

**Table 1:** Animal models utilized in each category

	Percentage
Genetically Engineered	
Mice	79%
Rat	14%
Monkey	7%
Xenograft	
Mice	93%
Monkey	7%
Disease Induction	
Monkey	37%
Rat	27%
Mice	18%
Rabbit	18%
Spontaneous	
Dog	50%
Cat	25%
Mice	25%

### Utilization of animal models in preclinical investigations of cancer-related products

Among the 74 scrutinized studies, 18 were pertinent to cancer-related products (Table 2). Notably, animal models predominated as a fundamental component of these investigations, with the xenograft methodology being the principal mode of model generation, encompassing 61% of cancer-related animal models. In contrast, the remaining 39% comprised 6% attributed to genetic engineering, and 33% either lacked explicit animal model descriptions or adopted unspecified models. A significant proportion of 67% featured mice as the primary animal model species. Additionally, monkeys were employed in 11% of the studies related to cancer, while a singular study employed guinea pigs. Remarkably, a subset of three studies within this domain dispensed together the use of animal models.

**Table 2:** Animal models utilized in preclinical studies of cancer-related products

Year of Approval	Trade name (General name)	Target cell (in vivo/ex-vivo	Indication	Animal model	Details	Comments	Category	References
2003	Gendicine	In vivo	Head and neck cancer (SCC)	mice	knockout	Conditional knockout mouse model	Genetically Engineered	[25, 26]
2005	Oncorine	In vivo	Nasopharyngeal carcinoma	guinea pigs	N/A	Injected with Oncorine at dose levels of 5.0 × 10^10^ TCID50/kg, 1.0 × 10^11^ TCID50/kg, or 2.0 × 10^11^ TCID50/kg subcutaneously	N/A	[27]
2005	Oncorine	In vivo	Nasopharyngeal carcinoma	mice	Immunodeficient	Nude—A dose dependent manner from 5 × 10^4^ to 4 × 10^7^ TCID50/mm^3^	N/A	[27]
2007	Rexin-G	In vivo	Soft tissue sarcoma and osteosarcoma	mice	Immunodeficient	A nude mouse model of liver metastasis and in a subcutaneous human xenograft model of pancreatic cancer	Xenograft	[28]
2015	Imlygic	In vivo	Melanoma	mice	Immunodeficient	Nude—BALB/c—Subcutaneous injection of 2 × 10^6^ of the appropriate tumor cells and tumors allowed to develop to an average diameter of approximately 0.5 cm	Xenograft	[29, 30]
2015	Imlygic	In vivo	Melanoma	mice	N/A	C57BL/6—Harding–Passey melanoma cells (1 × 10^6^) were injected subcutaneously in shaved areas of the flank, bilaterally	Xenograft	[29, 30]
2017	Kymriah	Ex-vivo	Relapsed B-cell acute lymphoblastic leukemia	mice	Immunodeficient	An immunodeficient NOD/Shi-scid IL-2Rγ null human leukemia xenograft mouse model—no lymphoma animal model was developed and tested as a proof of concept	Xenograft	[31, 32]
2017	Yescarta	Ex-vivo	Relapsed or Refractory large B-cell lymphoma	Without animal model	–	No lymphoma animal model was developed and tested as a proof of concept	–	[33]
2020	Tecartus	Ex-vivo	Relapsed/refractory mantle cell lymphoma	Without animal model	–	There are no representative in vitro assays, ex vivo models, or in vivo models	–	[34]
2021	Abecma	Ex-vivo	Multiple myeloma	mice	Immunodeficient	NSG mice with and without BCMA + xenografts	Xenograft	[35]
2021	ARI-0001	Ex-vivo	Adult relapsed/refractory acute lymphoblastic leukemia	mice	Immunodeficient	NOD/scid-IL-2Rnull—They were inoculated intravenously (i.v.) with 0.3 × 10^6^ GFP-NLuc Namalwa cells per mice	Xenograft	[36]
2021	Breyanzi	Ex-vivo	Relapsed or refractory diffuse large B-cell lymphoma; follicular lymphoma	mice	Immunodeficient	Raji xenograft athymic nude—No lymphoma animal model was developed and tested as a proof of concept. More information is given in the source	xenograft	[37, 38]
2021	Carteyva	Ex-vivo	Relapsed or refractory diffuse large B-cell lymphoma	N/A	N/A	No more information was found for this product	N/A	N/A
2021	Delytact	In vivo	Malignant Glioma	mice	Immunocompetent & athymic	Dissociated 005 GSCs (2–5 × 10^4^ cells) in 3 μL PBS were implanted stereotaxically into the striatum to generate orthotopic intracranial tumors	Xenograft	[39, 40]
2022	Adstiladrin	In vivo	Bladder cancer	mice	Immunodeficient	An orthotopic mouse model of human bladder cancer	Xenograft	[41]
2022	Adstiladrin	In vivo	Bladder cancer	monkey	N/A	No more information was found for this model	N/A	[41]
2022	Carvykti	Ex-vivo	Relapsed or refractory multiple myeloma	mice	Immunodeficient	NOG mice aged 6–8 weeks were injected s.c. with NCI-H929 cells (5 × 10^6^ cells) and tumor volume measured under blind conditions twice/week by caliper	Xenograft	[42]
2022	Carvykti	Ex-vivo	Relapsed or refractory multiple myeloma	monkey	N/A	i.v. injection of BCMA-TCB2	Xenograft	[42]

Within the realm of preclinical appraisals about the aforementioned products, cell line-derived xenograft (CDX) models were notably prominent, particularly in the context of bone marrow cancers. It is worth highlighting that nude or immunodeficient mice receiving cancer cell grafts constituted the most frequently employed animal species. Moreover, the products Carvykti and Oncorine uniquely involved the utilization of monkeys and guinea pigs, respectively. In the context of lymphoma, associated with five distinct products, namely, Carteyva, Breyanzi, Tecartus, Kymriah and Yescarta, a conspicuous deficiency in efficient animal models for lymphoma was observed. Consequently, the relevant documentation articulated the absence of animal studies conducted for lymphoma [33, 34, 37, 38]. However, in the case of Breyanzi, a noteworthy exception emerged, wherein despite the initially stated lack of an efficient model for lymphoma, pharmacological investigations were conducted employing a Raji xenograft animal model [37]. This model was fashioned based on a distinctive framework devised by Buchsbaum and colleagues [38], characterized by specific attributes. A solitary instance within this purview featured the application of a conditional knockout mouse model, exclusively pertinent to Gendicine. It is pertinent to note that the spectrum of animal models for this particular drug extends more comprehensively, albeit with limited available information drawn from recent studies [25].

### Utilization of animal models in preclinical investigations of nononcological products

Among the 74 scrutinized studies, 52 were directed toward nononcological products, encompassing a substantial proportion dedicated to genetic disorders (Table 3). In contrast to preclinical studies of cancer, 55% of the investigations in this section employed genetically engineered as the primary method for generating animal models. Induction techniques were applied in 17% of instances, while natural occurrences accounted for 8%, and xenografts represented 4%. The preeminent animal model employed in nononcological inquiries paralleled the cancer research sphere, with mice serving as the predominant choice, utilized in 53% of cases. In addition to mice, nonhuman primates featured more prominently, constituting 19% of the studies. Rats were also frequently enlisted, contributing to 16% of the animal models in this category. Other species enlisted in this realm comprised rabbits (4%), dogs (4%), and cats (2%).

**Table 3:** Animal Models Utilized in Preclinical Studies of Nononcological Products

Year of Approval	Trade name (General name)	Target cell (in vivo/ex-vivo	Indication	Animal model	Details	Comments	Category	References
2011	Neovasculgen	In vivo	Peripheral vascular disease and limb ischemia	rabbit	N/A	With bone grafting (n = 12) and empty defects (n = 6)	Disease induction	[43]
2012	Glybera	In vivo	Familial Lipoprotein Lipase Deficiency	cat	N/A	Naturally, homozygous for an LPLG412R mutation	Spontaneous	[44, 45]
2012	Glybera	In vivo	Familial Lipoprotein Lipase Deficiency	mice	Immunocompetent	LPL knockout mouse—used for initial studies	Genetically engineered	[44, 45]
2012	Glybera	In vivo	Familial Lipoprotein Lipase Deficiency	mice	Immunocompetent	LPL knockout mouse—further complicated by the presence of significant local muscle pathology	Genetically engineered	[44, 45]
2013	Kynamro	In vivo	Homozygous familial hypercholesterolemia	mice	N/A	Mice bred with no LDL receptor and expressing human apoB-100 who developed extensive atherosclerotic plaques	Genetically engineered	[46]
2016	Ampligen	In vivo	Chronic fatigue syndrome/myalgic encephalomyelitis	N/A	N/A	No information was found for this product	N/A	N/A
2016	Exondys 51	In vivo	Duchenne Muscular Dystrophy (DMD)	mice	*mdx*	Up to 960 mg/kg/week of IV-administered eteplirsen	genetically engineered	[47]
2016	Exondys 51	In vivo	Duchenne Muscular Dystrophy (DMD)	monkey	N/A	Cubcutaneous or IV administration of eteplirsen up to the 320 mg/kg maximum dose	N/A	[47]
2016	Spinraza	In vivo	Spinal Muscular Atrophy	mice	Δ7	The complete details pertaining to the model are provided within the source	Disease induction	[48]
2016	Spinraza	In vivo	Spinal Muscular Atrophy	mice	Taiwanese type I	The complete details pertaining to the model are provided within the source	Genetically engineered	[48]
2016	Spinraza	In vivo	Spinal Muscular Atrophy	mice	Taiwanese type III	The complete details pertaining to the model are provided within the source	Genetically engineered	[48]
2016	Spinraza	In vivo	Spinal Muscular Atrophy	mice	Burgheron	The complete details pertaining to the model are provided within the source	Genetically engineered	[48]
2016	Strimvelis	Ex-vivo	Severe combined immunodeficiency (SCID) due to ADA deficiency	mice	Immunodeficient	Peripheral blood lymphocytes from patients affected by ADA- SCID were transduced with a retroviral vector for human ADA and injected into immunodeficient mice	Xenograft	[49, 50]
2016	Zalmoxis	Ex-vivo	Restoring the immune system of the patient after hematopoietic stem cell transplantation	mice	Immunodeficient	NOD—Subcutaneously transplanted with human skin	Xenograft	[51]
2017	Invossa	Ex-vivo	Moderate Knee Arthritis	rat	MIA	Monosodium ModoAcetate + Surgery	Disease induction	[52, 53]
2017	Luxturna	In vivo	RPE65 mutation associated retinal dystrophy	dog	N/A	Naturally occurring animal model with mutated RPE65	Spontaneous	[54]
2018	Onpattro	In vivo	Hereditary Transthyretin-related Amyloidosis	mice	hTTR V30M HSF1^±^	The complete details pertaining to the model are provided within the source	Genetically engineered	[55]
2018	Tegsedi	In vivo	Hereditary Transthyretin-related Amyloidosis	mice	hTTR-Ile84Ser	The complete details pertaining to the model are provided within the source	Genetically engineered	[56, 57]
2019	Collategene	In vivo	Critical Limb Ischemia	rabbit	N/A	Surgical operation	Disease induction	[58, 59]
2019	Collategene	In vivo	Critical Limb Ischemia	rat	N/A	Surgical operation	Disease induction	[58, 59]
2019	Vyondys 53	In vivo	Duchenne Muscular Dystrophy	mice	*mdx/ulmr^* ~ */* *Xist^^*	The complete details pertaining to the model are provided within the source	Genetically engineered	[60, 61]
2019	Waylivra	In vivo	Adult Familial Chylomicronemia syndrome	mice	N/A	C57BL/6 mice, Ldlr -/- mice (B6.129S7-Ldlrtm1Her/J, Jackson Laboratories, Bar Harbor, ME), Ob/Ob (B6. Cg-Lepob/J) and apoC-III -/- mice (B6.129P2 Apoc3tm1Unc)	Disease induction	[62, 63]
2019	Waylivra	In vivo	Adult Familial Chylomicronemia syndrome	mice	*CETP Transgenic* *Ldlr -/- Mice*	They were generated by breeding the huCETP Tg animals with mice lacking a functional LDL receptor	Genetically engineered	[62, 63]
2019	Waylivra	In vivo	Adult Familial Chylomicronemia syndrome	monkey	N/A	Administration of a high fructose supplement	Disease induction	[62, 63]
2019	Waylivra	In vivo	Adult Familial Chylomicronemia syndrome	rat	N/A	Sprague Dawley—fed a high fructose diet	Disease induction	[62, 63]
2019	Waylivra	In vivo	Adult Familial Chylomicronemia syndrome	rat	*Zucker diabetic*	ZDF-Leprfa/Crl—Full information about the model is given in the source	Genetically engineered	[62, 63]
2019	Zolgensma	In vivo	Pediatric Spinal Muscular Atrophy	mice	*SMNΔ7*	In the SMA mice model (SMNΔ7 mice) a decreased mass of the left ventricle and decreased wall thickness putatively due to eccentric hypertrophy is observed	Genetically engineered	[64, 65]
2019	Zolgensma	In vivo	Pediatric Spinal Muscular Atrophy	monkey	*SMA*	Injection of scAAV9.CB.GFP in young cynomolgus monkeys	Genetically engineered	[64, 65]
2019	Zynteglo	Ex-vivo	Adult transfusiondependent ß thalassemia	mice	Immunodeficient	BB305 LVV-transduced mouse bone marrow cells (BMCs) immunodeficient, myeloablated mice	Genetically engineered	[66]
2020	Givlaari	In vivo	Porphyria	mice	T1/T2 AIP	Combined PB/DDC inductions were performed in male AIP mice—Full information about the model is given in the source	Genetically engineered	[67–69]
2020	Givlaari	In vivo	Porphyria	monkey	N/A	Naive Chinese—Full information about the model is given in the source	N/A	[67–69]
2020	Givlaari	In vivo	Porphyria	rat	AIP	PBGD knockdown—Full information about the model is given in the source	Genetically engineered	[67–69]
2020	Leqvio	In vivo	Primary hypercholesterolemia	monkey	N/A	Dedicated PD drug interaction studies have not been conducted in animals but the applicant performed a 13-week repeated dose toxicology study in Cynomolgus monkeys with coadministration of inclisiran (once monthly SC) and/or atorvastatin (orally daily)	Disease induction	[70, 71]
2020	Libmeldy	Ex-vivo	Metachromatic Leukodystrophy	mice	As^2–/–^ MLD	C57BL/6 & congenic C57BL/6 Ly45.1—As^2–/–^ MLD mice were bred in the H.S. Raffaele animal research facility by intercrossing the homozygous offspring of two carrier mice obtained by rederivation (embryo transfer) of As2–/– males with C57BL/6 females	Spontaneous	[72, 73]
2020	Oxlumo	In vivo	Primary hyperoxaluria type 1	mice	AGT-deficient null	Mutant mice lacking liver AGXTmRNA and protein	Genetically engineered	[74, 75]
2020	Oxlumo	In vivo	Primary hyperoxaluria type 1	monkey	N/A	Naive Chinese	N/A	[74, 75]
2020	Oxlumo	In vivo	Primary hyperoxaluria type 1	rat	N/A	Sprague–Dawley	N/A	[74, 75]
2020	Viltepso	In vivo	Duchenne Muscular Dystrophy	dog	*CXMD*	With frozen spermatozoa driven from a golden retriever	Spontaneous	[76]
2021	Amondys 45	In vivo	Duchenne Muscular Dystrophy	mice	*mdx*	The complete details pertaining to the model are provided within the source	Genetically engineered	[77, 78]
2021	Skysona	Ex-vivo	Juvenile Cerebral Adrenoleukodystrophy	mice	Immunodeficient	There are no animal models of CALD that recapitulate the human disease and could be used for demonstration of improvements in cerebral inflammation and demyelination. Brain engraftment of Lenti-D transduced CD34 + HSCs myeloablated immunodeficient mice in pivotal combined in vivo	Genetically engineered	[79, 80]
2022	Hemgenix	In vivo	Hemophilia B	mice	Knock-out	B6.129P2-F9^tm1Dws^ mouse model of Hemophilia B	Genetically engineered	[81, 82]
2022	Hemgenix	In vivo	Hemophilia B	monkey	N/A	No more information was found for this model	N/A	[81, 82]
2022	Rovtavian	In vivo	Hemophilia A	mice	Immunodeficient	Immune deficient Rag2 constitutive knockout mouse model (B6.129S6-Rag2tm1Fwa N12; Rag2-/-)	Genetically engineered	[83]
2022	Rovtavian	In vivo	Hemophilia A	mice	Immunodeficient	The hemophilia A knockout mouse crossed with a Rag2-/- mouse model (B6;129S-F8tm1Kaz/J x B6.129S6-Rag2tm1Fwa N12; Rag2-/- x FVIII-/-)	Genetically engineered	[83]
2022	Rovtavian	In vivo	Hemophilia A	monkey	N/A	Rhesus—No more information was found for this model	N/A	[83]
2022	Rovtavian	In vivo	Hemophilia A	monkey	N/A	Cynomolgus—No more information was found for this model	N/A	[83]
2022	Upstaza	In vivo	Aromatic L-amino acid decarboxylase (AADC) deficiency	mice	AADC deficiency	AadcS250F/S250F mice carry a conserved C890T base pair (S250F amino acid) mutation in the mouse Ddc gene. This mutation corresponds to a human C835T missense mutation associated with infantile Parkinsonism	Genetically engineered	[84]
2022	Upstaza	In vivo	Aromatic L-amino acid decarboxylase (AADC) deficiency	monkey	N/A	Models of Parkinson’s disease	Genetically engineered	[84]
2022	Upstaza	In vivo	Aromatic L-amino acid decarboxylase (AADC) deficiency	rat	N/A	Models of Parkinson’s disease	Genetically engineered	[84]
2023	Elevidys	In vivo	Duchenne muscular dystrophy	mice	*mdx*	The complete details pertaining to the model are provided within the source	Genetically engineered	[85]
2023	Elevidys	In vivo	Duchenne muscular dystrophy	rat	Dmd^mdx^	The complete details pertaining to the model are provided within the source	Genetically engineered	[86]
2023	Vyjuvek	In vivo	Dystrophic epidermolysis bullosa	mice	Immunodeficient	Colony of homozygous Col7a1flNeo mice, which are a strain expressing only 10% of the amount of murine type VII collagen found in normal mouse skin. For xenografting, NOD/SCID mice were used (NOD.CB17-PrkdcSCID/J mice; stock 001303; The Jackson Laboratory)	Xenograft	[86]

Significantly, a substantial portion of the models within this category was rooted in genetically engineered models. Such models in preclinical studies emanated from two principal avenues: procurement from commercial laboratories or in-house generation by researchers. Moreover, in some investigations, the primary model served as a foundation, inheriting genetic alterations from other genetically engineered models, or the foundational disease model emerged through the mating of two distinct genetically modified models (as observed in the EMA (European Medicines Agency) document for Rovtavian) [83]. Additionally, mice, rats, and nonhuman primates were the prevalent species subjected to genetic engineering, each bearing unique attributes pertinent to specific research objectives. In the majority of cases, animals exhibited specific genetic aberrations, albeit certain exceptions involved the use of highly immunodeficient mice, as exemplified in the Skysona study [79].

Beyond genetic engineering, induction, natural occurrences, and xenograft methods also found applicability within this category. The induction methodology was multifariously employed to replicate disorders such as adult familial chylomicronemia syndrome and ischemia or arteritis, accomplished through specialized dietary regimens or surgical procedures. Rat and monkey species constituted the primary subjects of experimentation within this domain, although mice and rabbits were sporadically incorporated. In the natural occurrence category, dogs emerged as the primary species of choice, with a solitary instance of cat utilization documented [44]. A noteworthy case, pertinent to the Libmeldy product, involved the creation of an animal model through the interbreeding of two species with naturally occurring disorders [72]. In contrast, the adoption of xenograft techniques was relatively limited in this category, with only three investigations resorting to this method. Notably, Vyjuvek and Strimvelis product research incorporated the grafting of cells bearing disease-related defects into severely immunodeficient mice [49, 86]. The study associated with the Zalmoxis product similarly employed this method to augment the immune system following the grafting of hematopoietic stem cells.

Of the 74 examined studies, 4 studies were concerned with products about infectious diseases (Table 4). In these infectious disease inquiries, the predominant animal models of choice encompassed nonhuman primates and rabbits, primarily induced through techniques such as induction.

**Table 4:** Animal models utilized in preclinical studies of products related to infectious diseases

Year of Approval	Trade name (General name)	Target cell (in vivo/ex-vivo	Indication	Animal model	Details	Comments	Category	References
1998	Vitravene	In vivo	Local treatment of cytomegalovirus retinitis in immunocompromised patients	Monkey	N/A	Systematic—Treated for every other week up to 3 months—Investigating the metabolites in liver, kidney, and plasma	N/A	[87–89]
1998	Vitravene	In vivo	Local treatment of cytomegalovirus retinitis in immunocompromised patients	Rabbit	N/A	Local—Monitoring for safety, also metabolism and elimination were investigated	N/A	[87–89]
2020	Spikevax	In vivo	COVID-19 vaccination	Monkey	N/A	Were injected intramuscularly with 10 μg or 100 μg in a 1 ml of 1 × phosphate-buffered saline (PBS) of the mRNA1273 vaccine	disease induction	[90]
2020	Comirnaty	In vivo	COVID-19 vaccination	Monkey	N/A	No more information was found for this product	disease induction	[91]

### Trending approaches in the development of animal models for investigative research

The preeminent method for establishing animal models in cancer research is notably the xenograft approach. Within the purview of xenograft studies, the CDX method stands as the ubiquitous choice. Indeed, the advent of CDX models followed the discernment of metastatic tendencies and their intricate association with the site of tumour cell inoculation in laboratory animals. These models hinge upon the subcutaneous or intravenous injection of human cancer cells into immunocompromised mice, a procedure readily achievable within the confines of a laboratory setting. CDX models have exhibited marked efficacy in the development of cytotoxic cancer therapies [92]. However, they have proven less efficacious when utilized for drugs targeting specific proteins [93]. The utility of CDX models is contingent upon the specific objectives of a study. Among their advantages are their suitability for investigating underlying mechanisms, cost-effectiveness, and expeditious development. Additionally, they prove instrumental in the assessment of nonspecific cytotoxic agents. Conversely, their limitations encompass the lack of heterogeneity within models generated through this method, the inability to undertake immunological investigations utilizing these models, and their sole composition of cancer cells, bereft of the rich tumour microenvironment [94, 95]. Notwithstanding these drawbacks, CDX models remain the favoured choice for preclinical studies and find extensive use in the majority of scrutinized cases. Furthermore, their utilization in diverse research domains has witnessed a substantial upsurge, underscoring their enduring popularity [96].

It is imperative to also consider the emergence of patient-derived xenograft (PDX) models, which ameliorate the constraints intrinsic to other methodologies, yielding more efficacious animal models. PDX models preserve not only the tumour microenvironment but also the heterogeneity and mutagenic characteristics of tumours. Furthermore, they facilitate the study of metastasis, with the generated model serving as a suitable biological surrogate. However, it is noteworthy that PDX models can only be generated in severely immunocompromised mice, and their efficiency exhibits variability, rendering them less suitable for early-stage cancer research [97, 98]. Thus, a judicious evaluation of the facets of preclinical studies can lead to the adoption of novel and more efficacious models, enhancing the quality of such investigations.

Additionally, as previously mentioned, genetic manipulation has emerged as the preeminent method in investigations of nononcological diseases. This approach affords the potential for creating models that closely mirror the characteristics of the original disease. Recent years have witnessed a substantial proliferation in the usage of such models, attributed to the advent of engineered endonucleases, which enable precise and efficient genome editing [99–101]. The key step in genome editing is the induction of site-specific double-strand breaks (DSBs) by engineered endonucleases that are subsequently corrected by one of two competing DNA repair pathways, nonhomologous end-joining (NHEJ) and homology-directed repair (HDR) [102]. Recent advances in genome editing technologies reflect the rapid development of engineered endonucleases, including zinc finger nucleases (ZFNs), transcription activator-like effector nucleases (TALENs), and clustered regularly interspaced short palindromic repeat (CRISPR) systems [103]. These endonucleases endow genome editing with two pivotal attributes: 1) the capacity to selectively recognize specific target sequences and 2) a high degree of compatibility for the placement of specified sequences [104]. Predominantly, the genetic modifications affecting the animal models under scrutiny are knockouts. For instance, in a preclinical study centred on Glybera, a product related to familial lipoprotein lipase deficiency, mice with knockout genomic regions linked to lipoprotein lipase were employed [44]. Similarly, in the context of the Rovtavian product, which is associated with hemophilia A, knockout mice have been instrumental [83]. Such instances abound in the corpus of examined research.

The primary objective of knockout is to supplant a specific genomic segment with one that is either nonfunctional, modified, or irrelevant. This substitution can precipitate alterations in the phenotype of the animal model, thereby manifesting unique disease characteristics. The development of these models represents a watershed moment in the realm of animal models and therapeutic product development. The field has witnessed a plethora of advances that permit increasingly specific and temporally controlled genetic manipulations, in addition to confining mutations to designated tissues [105]. Notwithstanding these commendable strides, challenges persist in the handling of these models. For instance, target genes may not always be amenable to genetic manipulation, and genetic editing in these models is a complex endeavour that may engender metabolic perturbations within the animal’s pathways, precipitating phenotypic anomalies [106]. Nonetheless, the usage of genetically modified animal models is burgeoning, with the advent of novel technologies that hold the potential to ameliorate the limitations of prior models, thereby engendering models of greater aptitude than their predecessors.

### Trending species in the animal models for investigative research

As indicated by the findings of this study, the preclinical investigation of gene therapy products predominantly employs the mouse model, which stands as the most prevalent species of choice. Furthermore, upon closer scrutiny, it becomes evident that mice are extensively employed in the development of genetically modified animal models. The utilization of mice as an animal model boasts several merits, including cost-effectiveness in maintenance. In addition, their rapid reproduction rate and comparatively short lifespan render them ideal for genetic inquiries. Significantly, mice exhibit an estimated genetic similarity to humans in the range of 99% [107]. Furthermore, the extensive research conducted on their genetic resources, which are publicly accessible [108, 109], underscores their prominence as a preferred model for conducting preclinical investigations.

Consequently, following mice, nonhuman primates emerge as the second most utilized species in the research endeavours under review. Phylogenetically, nonhuman primates share the closest genetic proximity to humans and find widespread application in diverse domains, encompassing psychiatric, metabolic, reproductive, and immunological studies [52]. In the specific context of the studies under consideration, nonhuman primates were predominantly deployed for disease induction purposes. However, some instances featured their deployment as noncompliant subjects, likely chosen for safety and toxicity assessments. It is worth noting that despite the marked desirability of employing this species, limitations such as restricted availability, associated expenses, and ethical concerns regarding genetic manipulation serve as constraining factors [110].

Within the third category of animal models, rats were also included. Rats serve as apt animal models extensively employed in the examination of physiology and pathophysiology, and they constitute a suitable choice for evaluating the efficacy and toxicity of clinical trials [111–113]. In the studies scrutinized, rats were most frequently employed in genetic manipulations.

Last, it is noteworthy that dogs were solely featured in the studies under consideration as models with naturally occurring traits. Specifically, hereditary diseases in dogs, classified as naturally occurring, bear the highest clinical resemblance to human diseases [114]. This congruence has engendered substantial demand for the use of dogs in these particular contexts.

## Conclusions

The selection of an appropriate animal model constitutes a pivotal and fundamental step in the execution of animal studies, particularly within the domain of preclinical research. This selection process necessitates strict adherence to established scientific criteria and standards, as it holds the key to attaining optimal outcomes not only in the present investigation but also in subsequent research endeavours. An effective strategy for model selection involves recourse to prior studies that have traversed all requisite phases, culminating in the approval of resultant products. By doing so, one can confidently employ the chosen animal model and extend the generalizability of its findings to forthcoming investigations. Moreover, this retrospective approach enables the identification of successful methodologies for generating animal models and the identification of species suitable for the intended research purposes.

In the context of the current study, we focused on the examination of animal models employed in preclinical assessments of gene therapy products. Our findings have illuminated that the xenograft methodology, predominantly implemented through the CDX technique, stands as the most prevalent approach in preclinical studies about cancer therapeutics. Furthermore, in the realm of generating animal models for diverse pathologies, with a particular emphasis on genetic disorders, genetic manipulation emerges as the predominant technique, particularly in the creation of knockout models. Within this landscape, mice and nonhuman primates have emerged as the two most frequently utilized species.

Notably, recent trends underscore a discernible upswing in the utilization of mice and genetic manipulation methodologies as we approach the contemporary era. It is imperative not to overlook the transformative potential inherent in emerging technologies for the creation of these animal models, as the incorporation of state-of-the-art innovations undoubtedly holds promise for the generation of models of superior quality and fidelity.

## Data Availability

All datasets on which the conclusions of this article rely are presented within the article. No additional data repositories are required as all relevant data can be found within the manuscript itself. We have taken care to ensure that the data is easily accessible to readers in the main paper.

## Abbreviations

NRC: National research council
CDX: Cell line-derived xenograft
EMA: European medicines agency
PDX: Patient-derived xenograft
DSBs: Double-strand breaks
NHEJ: Nonhomologous end-joining
HDR: Homology-directed repair
ZFNs: Zinc finger nucleases
TALENs: Transcription activator-like effector nucleases
CRISPR: Clustered regularly interspaced short palindromic repeat
